# Analysis of the onset of lower urinary tract symptoms in multiple scleroris patients

**DOI:** 10.1007/s00345-025-05709-y

**Published:** 2025-05-31

**Authors:** Jan Krhut, Pavel Hradílek, Adéla Kondé, Barbora Skugarevská, Ivana Woznicová, Radek Paus Sýkora, Hanne Kobberø, Tomáš Rychlý, Peter Zvara

**Affiliations:** 1 Department of Urology, University Hospital, Tř. 17. listopadu 1790, Ostrava, 708 52 Czech Republic; 2 Department of Surgical Studies, Ostrava University, Ostrava, Czech Republic; 3 Department of Neurology, University Hospital, Ostrava, Czech Republic; 4 Department of Clinical Neurosciences, Ostrava University, Ostrava, Czech Republic; 5 Department of Applied Mathematics, Faculty of Electrical Engineering and Computer Science, VSB - Technical University, Ostrava, Czech Republic; 6 Department of the Deputy Director for Science, Research and Education, University Hospital, Ostrava, Czech Republic; 7 Department of Urology, Odense University Hospital, Odense, Denmark; 8 Department of Regional Health Research, University of Southern Denmark, Odense, Denmark

**Keywords:** Lower urinary tract symptoms, Multiple sclerosis, Urgency, Incontinence

## Abstract

**Purpose:**

To estimate the timepoint of onset of the lower urinary tract symptoms (LUTS) over the course of multiple sclerosis (MS), determine which of the LUTS typically appears first, and identify risk factors for early development of LUTS in patients with MS (PwMS).

**Methods:**

This observational study included 1167 PwMS. The participants were predominantly women (72%), median age was 45 (IQR 36;53) years, and median disease duration was 11 (IQR 6;16) years. Inclusion criteria were age over 18 years, proven MS diagnosis according to McDonald´s criteria (2017), and complete medical records since establishment of MS diagnosis. A structured in-person interview addressing the study objectives was performed during routine follow-up visit. The data were cross-checked with the medical records.

**Results:**

Median LUTS-free survival time after MS diagnosis has been made, was 8 (IQR 7;10) years. Storage, voiding and postmicturition symptoms were identified as a first LUTS in 549/709 (77%), 130/709 (18%), and 30/709 (4.2%) PwMS, respectively. Most frequently reported first LUTS was urgency 360 (51%). Using multivariate analysis, higher age, higher degree of disability, and presence of cerebellar and/or pyramidal symptoms at the time of MS diagnosis were significantly associated with shorter LUTS-free survival. No significant relationship between LUTS-free survival and sex or type of MS was found.

**Conclusions:**

Our data show, that LUTS occur after median time of 8 years after MS diagnosis, with urgency being the most frequently reported first LUTS.

## Introduction

Multiple sclerosis (MS) is a chronic demyelinating, inflammatory, degenerative disease with increasing prevalence. About 2.8 million individuals are affected worldwide [1]. MS poses significant burden on individuals and society, especially because young active people are often affected [2].

Together with fatigue, impairment of mobility and sensitivity, balance and coordination disturbances, blurred vision, depressions, cognitive impairment and impairment of bowel function, lower urinary tract symptoms (LUTS) represent one of the most common consequences of MS. A high prevalence of LUTS in patients with multiple sclerosis (PwMS) was first noted by Oppenheim in the late 19th century [3]. LUTS significantly impact daily life of affected individuals by limiting their social activities, travel, and work performance [4]. Available studies estimated the prevalence of LUTS in PwMS between 32% and 96.8% [5]. This wide range may be attributed to the cross-sectional nature of most studies with highly heterogeneous characteristics with respect to age, disease type, duration and degree of disability [6, 7]. Given the progressive character of MS, it is assumed that LUTS develop in substantial proportion of PwMS, however exact data on the time course of their onset are lacking [8].

Therefore the primary goal of this study was to determine the timepoint of onset of the LUTS over the course of MS. In addition, we aimed to determine which of the LUTS typically appears first and what are the risk factors for early development of LUTS.

## Materials and methods

### Protocol approvals and registrations

Study was performed in accordance with Declaration of Helsinki, World Health Organization. The study protocol was approved by Institutional Review Board and registered on ClinicalTrials.gov as NCT-06226831. The study is reported according to STROBE guidelines [9].

### Study design and population

This non-interventional retrospective observational study was conducted at a single regional Multidisciplinary Center for Diagnostics and Treatment of MS (Center). This Center is responsible for diagnostics, treatment and follow-up of all PwMS in its catchment area with population of 1.2 million. The primary endpoint was the timepoint of onset of the LUTS over the course of MS. Secondary endpoints included determination of LUTS which developed first, and identification of risk factors for early development of LUTS.

Eligible subjects were identified using detailed search in the database of the Center. This database provides access to medical records of all consecutive patients, who visited the Center, at least once, starting from 1996 until the search has been performed between March and May 2019. Initial database search based on inclusion criteria of proven MS diagnosis according to McDonald´s criteria (2017) [10] and age over 18 years, identified 2348 individuals. A total of 662 patients who were lost to follow-up (*n* = 359), died (*n* = 178) or moved (*n* = 125) were excluded. Remaining 1686 PwMS gave informed consent and were enrolled into the study. During their routine follow-up appointment at the Center between June 2019 and June 2023, a structured in-person interview with the patient was performed by a physician. During this interview, patients were provided with a list of LUTS along with explanations comprehensible to lay person. They were subsequently asked if they experienced any of the symptoms, which symptom appeared first, and when it occurred in relation to the initial MS diagnosis. The data from this interview were cross-checked with medical records of the individual patient which included *the* Expanded disability status scale (*EDSS) total score * [11] *and sub-scores for individual subdomains*, *including bladder symptoms*. The fact that EDSS has been recorded at each visit at the Center, allowed us to determine the time point of onset of LUTS. *In case of discrepancy between the patient’s recall and the medical records*, *the patient was excluded from the final analysis (n = 519)*. Along with demographic data and clinical characteristics relevant to the time of data collection, we gathered information on type of MS, the presence of cerebellar and/or pyramidal symptoms (CPS), the presence of oligoclonal bands (OCB) in the cerebrospinal fluid, disability level, as measured by the EDSS, and other variables corresponding to the time of diagnosis of MS.

### Statistical analysis

Numerical variables (i.e. age at different time points of examined period, follow-up, and EDSS) are expressed as medians and the interquartile ranges (IQR). Categorical variables (i.e. sex, type of MS, occurrence of CPS as the first manifestation of MS, presence of OCB in the cerebrospinal fluid at MS diagnosis, and occurrence of LUTS at data collection) are presented as the absolute frequencies and relative frequencies (%) with 95% Clopper-Pearson confidence intervals (95%CI), if applicable. Cox proportional-hazards model was used to assess the relationship of LUTS-free survival and selected risk factors. Based on the literature, sex, type of MS, ocurrence of CPS as the first manifestation of MS, occurrence of OCB in cerebrospinal fluid at MS diagnosis, age and EDSS at MS diagnosis were selected for the risk factors [12, 13]. LUTS-free survival was visualized with the Kaplan-Meier curve. All statistical analyses were performed using R software (version 4.4.1, www.r-project.org) and the significance level was set to 0.05.

## Results

In total, 1167 PwMS were included in the final analysis. The demographic and clinical characteristics of the cohort are shown in Table 1.

**Table 1:** Demographic and clinical characteristics (*n* = 1167)

	Median (IQR) or *n* (%)
Age at data collection (years)	45 (36;53)
Sex	
Female	835 (72)
Male	332 (28)
Type of MS at data collection	
Relapsing-remitting	1056 (90)
Progressive	111(10)
CPS at MS diagnosis	
Yes	754 (65)
No	413 (35)
OCB at MS diagnosis	
Yes	1111 (95)
No	56 (5)
LUTS at data collection	
Yes	709 (61)
No	458 (39)
Age at MS diagnosis (years)	32 (26; 40)
EDSS at MS diagnosis	2.0 (1.5; 3.0)
Time from the MS diagnosis to data collection (follow-up, years)	11 (6; 16)

The values represent the median and the interquartile range (IQR) or the absolute and relative frequencies (%)

IQR: interquartile range, MS: multiple sclerosis, CPS: presence of cerebellar and/or pyramidal symptoms as the first manifestation of MS, OCB: presence of oligoclonal bands in the cerebrospinal fluid at time of MS diagnosis, LUTS: lower urinary tract symptoms, EDSS: Expanded disability status scale

The median MS duration in our cohort was 11 (6;16) years and the prevalence of LUTS was 709/1167 (61%, 95%CI: 58–64%). Storage, voiding and postmicturition symptoms were reported to be first LUTS in PwMS in 549/709 (77%, 95%CI: 74–80%), 130/709 (18%, 95%CI: 16–21%), and 30/709 (4.2%, 95%CI: 2.9–6.0%), respectively. Most frequently reported first LUTS was urgency 360/709 (51%, 95%CI: 47–55%). Detailed distribution of the first LUTS is presented in Table 2.

**Table 2:** Type of first LUTS in PwMS who have developed LUTS prior to the time of data collection (*n* = 709)

	Absolute frequency	Relative frequency %(95%CI)
**Storage symptoms**	**549**	**77 (74; 80)**
Urgency	360	51 (47; 55)
Frequency	76	11 (8.5; 13)
Urgency urinary incontinence	67	9.4 (7.4; 12)
Stress urinary incontinence	26	3.7 (2.4; 5.3)
Nocturia	20	2.8 (1.7; 4.3)
**Voiding symptoms**	**130**	**18 (16; 21)**
Straining	51	7.2 (5.4; 9.3)
Hesitancy	50	7.1 (5.3; 9.2)
Intermittent stream	21	3.0 (1.8; 4.5)
Slow stream	8	1.1 (0.48; 2.2)
**Postmicturion symptoms**	**30**	**4.2 (2.9; 6.0)**
Feeling of incomplete emptying	24	3.4 (2.2; 5.0)
Postmicturion dribble	6	0.85 (0.31; 1.8)

The values represent absolute and relative frequencies (%) supplemented by 95% confidence intervals (95%CI)

LUTS: lower urinary tract symptoms

**Fig. 1:**
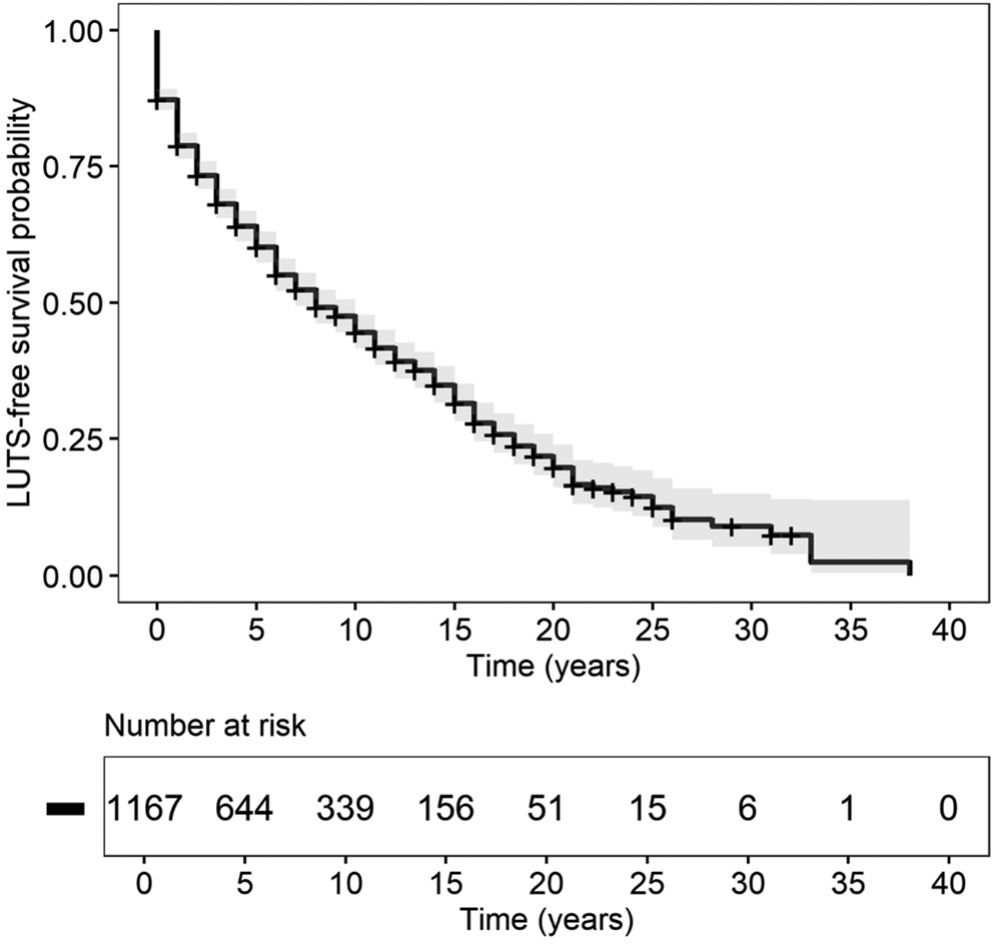
Kaplan-Meier curve showing the LUTS-free survival (*n* = 1167). The grey area represents the 95% confidence intervals around the Kaplan-Meier curve. Patients were censored, if they did not experience any LUTS at the point of data collection. LUTS: lower urinary tract symptoms

Median LUTS-free survival time after MS diagnosis was 8 (7; 10) years. Notably, 5-, 10-, and 20-year LUTS-free survival rate was 60%, 45%, and 20%, respectively. LUTS-free survival rate dropped below 10% after 26 years from MS diagnosis. The results are presented in Fig. 1.

Multivariate analysis of risk factors including sex, type of MS, presence of CPS at MS diagnosis, presence of OCB in cerebrospinal fluid at MS diagnosis, age at MS diagnosis and EDSS at MS diagnosis was conducted. Higher age, higher EDSS, presence of CPS and occurrence of OCB in cerebrospinal fluid at MS diagnosis were significantly associated with shorter LUTS-free survival. No significant relationship between LUTS-free survival and sex or type of MS was found. The results are presented in Table 3.

**Table 3:** Multivariate analysis using Cox proportional-hazards model for LUTS-free survival time

	HR (95%CI)	*p*
Sex		
Female	Reference	---
Male	1.13 (0.96; 1.34)	0.1
Type of MS		
Progressive	Reference	---
Relapsing-remitting	1.07 (0.83; 1.37)	0.6
CPS at MS diagnosis		
Yes	1.27 (1.07; 1.51)	0.007
No	Reference	---
OCB at MS diagnosis		
Yes	1.46 (1.04; 2.05)	0.027
No	Reference	---
Age at MS diagnosis	1.03 (1.02; 1.04)	< 0.001
EDSS at MS diagnosis	1.27 (1.19; 1.36)	< 0.001

The values represent the hazard ratio (HR), its 95% confidence interval (95%CI) and the *p*-value of Wald test

MS: multiple sclerosis, CPS: occurrence of cerebellar and/or pyramidal symptoms at the time of MS diagnosis, OCB: oligoclonal bands in the cerebrospinal fluid at the time of the diagnosis, EDSS: Expanded disability status scale, LUTS: lower urinary tract symptoms

## Discussion

LUTS represent one of the most bothersome manifestation of MS. Despite the high prevalence and proven negative impact on the quality of life (QoL), LUTS in PwMS are often overlooked and therefore not adequately treated [14, 15].

Our data indicate that the median time between MS diagnosis and onset of the first LUTS was eight years. This aligns with findings reported by Betts et al. This is the only paper that we identified which directly assessed, among other variables, onset of LUTS in a cohort of 170 PwMS [16]. The often cited review on neurogenic lower urinary tract dysfunctions in PwMS by de Seze et al. suggested that LUTS typically appear, on average, six years after onset of the disease, with the range varying from 5 to 9.5 years. However, none of the studies cited in this review, which formed the basis of this conclusion, specifically evaluated the time of onset of LUTS in a large group of PwMS [17].

Our results documented that urgency and other storage symptoms are the most frequently reported first LUTS in PwMS. While not directly addressed in this study, other have shown that urgency and urinary incontinence lead to most substantial impairment of both general and disease-specific QoL [18]. Early diagnosis and initiation of treatment of lower urinary tract dysfunction (LUTD) can relieve LUTS, significantly improve patients’ QoL, and prevent secondary complications including urinary tract infection, upper tract dilatation, urolithiasis, vesicoureteral reflux, and, in rare cases, renal failure [19]. These facts highlight the importance of considering LUTS during diagnostic work-up and the role of the urologist within the multidisciplinary team caring for PwMS.

All medical care for PwMS in Czech Republic takes place at the network of 15 specialized centers. Upon suspicion of MS, all patients are referred to one of these centers for confirmation of the diagnosis. It is mandatory that the initial examination in the center is performed within three months from the time when patient with symptoms suspicious of MS is identified. Only these centers are authorized to confirm the final MS diagnosis, prescribe disease modifying treatment and perform the follow-up. This means that our patient population represents cross-section of all PwMS in the chatchment area of our Center and not just the most severe cases. Along with neurologists, other medical specialists including opthtalmologist, immunologist, gastroenterologist, gynecologist, psychiatrist, psychologist and physical therapist are involved. At the point of detection of first LUTS, the patient is referred to the urologist specialized in neurogenic LUTD who is responsible for diagnostics and treatment of LUTS according to the current guidelines [20].

To our knowledge, this is the first study with one of the endpoints to identify risk factors of early LUTS onset. Based on our observation we conclude that the typical PwMS at high risk of early onset of LUTS has higher age and higher EDSS at the time of the MS diagnosis and presents early with CPS. In contrast, younger patients with lower EDSS score without CPS at the time of MS manifestation are at lower risk of early LUTS development. Importantly, the progressive type of MS was not identified as a risk factor for early onset of LUTS. This finding was unexpected. Proportion of patients with progressive MS in our cohort at the time of data collection was 10%, which is in agreement with current literature [21]. However given the progressive nature of MS, disease type might have changed between LUTS onset and the time of data collection. This could have biased the risk factor analysis with respect to this respective parameter. The fact that we did not show that patients with progressive disease are at higher risk of early onset of LUTS does not exclude possibility that LUTD in these patients could be more severe and subsequently lead to secondary complications. Closer urological follow-up of these PwMS is warranted.

Our findings are generally consistent with other studies that report correlation between the presence of LUTS and the extent of MS-related disability, as well as evidence of pyramidal symptoms in the lower limbs [22]. Several studies have also identified correlation between presence of LUTS and demyelinating lesions in specific brain and spinal cord structures as detected by MRI scan. Pontine lesions are suggested to be associated with incontinence and a weak urinary stream, whereas lesions in the cervical spinal cord appear to be linked with detrusor-sphincter dyssynergia [23–25]. We did not include lesion topography in the risk factor analysis for early LUTS onset in this study. This decision was based on the fact that majority of patients present with lesions in multiple locations on their initial MRI scans. This precludes analysis of correlation between lesion location, size, and the time of onset of LUTS.

In our cohort, with median MS duration of 11 years, the overall LUTS prevalence was 61%. This is consistent with data from a recently published meta-analysis, which reported LUTS prevalence in PwMS of 68.4% and 63.5% using self-reported and objective measures, respectively [26].

Importantly, our findings indicate that, after a certain duration of MS, nearly all patients will develop LUTS. This prediction is further supported by our analysis, which shows that the LUTS-free survival rate among patients with MS lasting more than 30 years is close to 0%. Notably, we assessed solely presence or absence of LUTS, not the degree of bother they caused to the individual patient.

In total, 17 patients reported that their LUTS have developed prior to any neurological symptoms atributable to MS. In these patients LUTS might represent the first manifestation of MS. However, since these recall data could not have been cross-checked with the medical records (no existing records available in the Center database prior to establishment of MS diagnosis), according to the study protocol, these patients were not included in the final analysis and reported in results.

This study is based on self–assessment of the LUTS by PwMS. Symptoms alone however do not provide objective information about the function of the lower urinary tract and are not sufficient to make a diagnosis with respect to the underlying pathology. This implicates that diagnostics and treatment of LUTS should be based on appropriate diagnostic algorithm [27].

Strenghts of this study include high number of enrolled subjects and multidisciplinary team of investigators. Several limitations have to be acknowledged. Those include retrospective design of the study and our inability to exclude LUTS which developed due to other comorbidities or other confounding factors. The probability that these factors affected significantly overall accuracy is reduced by a low median age of PwMS at onset of LUTS in our cohort. In addition, large rate of exclusion could pose selection bias. However, we suggest that the fact that 44% of patients were excluded due to discrepancy between their recall and the information in their medical records could have contributed to the validity of our results. Another argument supporting the consistency of our data is the fact, that the prevalence of LUTS and distribution of individual types of LUTS are similar to those previously reported [26, 28].

## Conclusion

Our data suggest, that LUTS occur after median time of 8 years after MS diagnosis, with urgency being the most frequently reported first LUTS. We identified the higher age, higher EDSS, and presence of cerebellar and/or pyramidal symptoms at the time of the MS diagnosis, as risk factors for early onset of LUTS. Appropriate diagnostics and treatment of LUTS may significantly contribute to the improving of QoL and prevent late complicactions in PwMS.

## Data Availability

No datasets were generated or analysed during the current study.

## Abbreviations

CPS: Cerebellar and/or pyramidal symptoms
EDSS: Expanded disability status scale
IQR: Interquartile range
LUTS: Lower urinary tract symptoms
MS: Multiple scleroris
OCB: Oligoclonal bands
PwMS: Patients with multiple sclerosis
STROBE: Strengthening the reporting of observational studies in epidemiology
